# The CD200/CD200R signaling pathway contributes to spontaneous functional recovery by enhancing synaptic plasticity after stroke

**DOI:** 10.1186/s12974-020-01845-x

**Published:** 2020-05-30

**Authors:** Hao Sun, Xinran He, Xia Tao, Tingting Hou, Mingming Chen, Meijun He, Hong Liao

**Affiliations:** Jiangsu Key laboratory of Drug Screening, China Pharmaceutical University, 24 Tongjiaxiang Street, Nanjing, 210009 China

**Keywords:** CD200/CD200R, Stroke, Inflammation, Spontaneous recovery, Synaptic plasticity

## Abstract

**Background:**

Spontaneous functional recovery occurs during the acute phase after stroke onset, but this intrinsic recovery remains limited. Therefore, exploring the mechanism underlying spontaneous recovery and identifying potential strategies to promote functional rehabilitation after stroke are very important. The CD200/CD200R signaling pathway plays an important role in neurological recovery by modulating synaptic plasticity during multiple brain disorders. However, the effect and mechanism of action of the CD200/CD200R pathway in spontaneous functional recovery after stroke are unclear.

**Methods:**

In this study, we used a transient middle cerebral artery occlusion (MCAO) model in rats to investigate the function of CD200/CD200R signaling in spontaneous functional recovery after stroke. We performed a battery of behavioral tests (Longa test, adhesive removal test, limb-use asymmetry test, and the modified grip-traction test) to evaluate sensorimotor function after intracerebroventricular (i.c.v.) injection with CD200 fusion protein (CD200Fc) or CD200R blocking antibody (CD200R Ab) post-stroke. Density and morphology of dendritic spines were analyzed by Golgi staining. Microglia activation was evaluated by immunofluorescence staining. Western blot was used to detect the levels of protein and the levels of mRNA were measured by qPCR.

**Results:**

Our study demonstrated that sensorimotor function, synaptic proteins, and structures were gradually recovered and CD200R was transiently upregulated in ipsilateral cortex after stroke. Synapse-related proteins and dendritic spines were preserved, accompanied by sensorimotor functional recovery, after stereotaxic CD200Fc injection post-stroke. In addition, CD200Fc restrained microglia activation and pro-inflammatory factor release (such as *Il-1*, *Tnf-α*, and *Il-6*) after MCAO. On the contrary, CD200R Ab aggravated sensory function recovery in adhesive removal test and further promoted microglia activation and pro-inflammatory factor release (such as *Il-1*) after MCAO. The immune-modulatory effect of CD200/CD200R signaling might be exerted partly by its inhibition of the MAPK pathway.

**Conclusions:**

This study provides evidence that the CD200/CD200R signaling pathway contributes to spontaneous functional recovery by enhancing synaptic plasticity via inhibition of microglia activation and inflammatory factor release.

## Introduction

Stroke, among which ischemic stroke accounts for nearly 87%, is a leading cause of death and disability worldwide, causing ~ 80% of all disabilities [1]. Due to improved surgical interventions, an increasing number of patients survive the devastating initial event but suffer from ongoing sensorimotor and cognitive dysfunction [2, 3]. Despite immediate and long-lasting spontaneous functional recovery, the degree of recovery remains limited and varies among individuals [3]. Spontaneous recovery involves a variety of cellular and molecular processes, and many restorative therapies depend on the processes observed during spontaneous recovery [4]. Therefore, it is important to investigate the mechanisms underlying spontaneous recovery and identify rehabilitative therapies to augment sensorimotor functional recovery. Such investigations will also improve our understanding of the pathological development of stroke.

The integrity of neuronal networks is the basis of sensorimotor behavior; however, neuronal networks and synapse circuits are damaged by stroke in regions that receive reduced blood supply, which results in sensorimotor dysfunction [5]. The surviving neural networks are partially rewired, and intact synapses are recruited during spontaneous recovery [6]. Thus, replacing the destroyed circuit and promoting surviving neural networks to remap might be treatment strategies to improve functional recovery after stroke.

Inflammation plays an important role in regulating synaptic plasticity [7–9]. Activated microglia are characterized as proinflammatory or anti-inflammatory microglia; microglia activation occurs within hours after stroke, and several pro-inflammatory factors (such as *Il-1*, *Tnf-α*, and *Il-6*) can be released by microglia in response to synapse degeneration or loss [10, 11]. Thus, inhibiting microglia activation and pro-inflammatory factor release may enhance synaptic plasticity and promote long-term stroke recovery. The CD200/CD200R signaling pathway has a unique expression profile, and it plays a profound regulatory role during neurological recovery under multiple brain pathological conditions. CD200, a member of the immunoglobulin superfamily, is widely expressed in neurons, astrocytes, and oligodendrocytes, whereas its receptor (CD200 receptor 1, CD200R1) is expressed in myeloid cells and microglia (the brain-resident myeloid cells) in rodents [12] and also highly expressed by neurons in human according to the Ben Barres database. The interaction between CD200 and CD200R1 is critical for inhibiting microglial activation and localized neuroinflammation during the pathological development of several brain diseases, including Parkinson’s disease, optic nerve crush, and germinal matrix hemorrhage [13–15]. The CD200/CD200R signaling pathway also participates in synaptic plasticity. Suppressing CD200/CD200R signaling via genetic approaches (deleting *Cd200*) impairs long-term potentiation (LTP) [16], whereas pharmaceutical approaches (using CD200Fc, a CD200R agonist) that activate CD200/CD200R signaling significantly enhance synaptic plasticity in AD and aged mice by regulating inflammation [17–19]. However, the role of the CD200/CD200R signaling pathway during spontaneous functional recovery remains unclear.

Here, we propose that the CD200/CD200R signaling pathway contributes to spontaneous functional recovery after stroke by restraining synapse loss through inhibiting microglia activation and pro-inflammatory factor release.

## Methods

### Animals

Adult male Sprague-Dawley (SD) rats (280–320 g, 8–10 weeks of age) were purchased from Shanghai Sippr-BK laboratory animal Co. Ltd. (Shanghai, China) and housed in the Pharmaceutical animal experimental center of China Pharmaceutical University under a 12 light–dark cycle with food and water ad libitum. All animal procedures were approved by the Animal Research Ethics Committee of China Pharmaceutical University.

### Animal surgery and drug administration

A transient right middle cerebral artery occlusion (MCAO) stroke was performed as previously described [20]. Briefly, rats were anesthetized with 2 % isoflurane, then exposing the right common carotid artery of rats. A 3-0 poly-lysine-coated monofilament nylon suture was inserted into the right internal carotid artery through the external carotid artery stump to occlude the middle cerebral artery. Two hours later, the filament was withdrawn to restore blood flow. Warming pads were used to maintain the body temperatures of animals at 37.0 ± 0.5 °C, and Doppler flowmetry (Moor Instruments, Essex, UK) was used to monitor cerebral blood flow (CBF) during the surgical procedure. In the ischemia phase, cerebral blood flow < 25% of the baseline was considered as successful ischemia.

The animals were anesthetized with 2 % isoflurane and placed on a stereotaxic apparatus (RWD Life Science, Shenzhen, China). After exposing the skull, a small hole was made to allow the intracerebroventricular injection. CD200Fc (5 μL, 4 μg/μL) (Jiangsu Futai Biotechnology, Taizhou, China) as an agonist of CD200R1, anti-CD200R1 antibody (CD200R Ab) (AbD Serotec, Oxford, UK) as an antagonist of CD200R1 (5 μL), or IgG (Santa Cruz Biotechnology Inc, USA) was injected into the right cerebral ventricle one time by a 10-μL Hamilton syringe 24 h after MCAO. The injection was controlled at a rate of 0.5 μL/min and the syringe remained in place for 5 min before the completion of injection. The stereotaxic coordinates of the right cerebral ventricle were as follows: bregma − 0.8 mm; lateral − 1.5 mm; ventral − 4.0 mm.

### Behavioral tests

Behavioral tests were performed at 4, 7, 14, 21, and 28 days after MCAO by an investigator blinded to the experimental groups.

#### Longa test

This test was assessed using a 5-point scale as described previously [20]: 0, no observable deficits; 1, failure to extend the left forepaw; 2, circling to the left; 3, falling to the left; and 4, unable to move spontaneously.

#### Adhesive removal test

To measure the sensory functions and motor functions, the tests were performed as described previously [21] with some modifications. The time that rats contacted and removed the spot was recorded and within a limit of 180 s.

#### Limb-use asymmetry test (cylinder)

To evaluate forelimb-use asymmetry, rats were placed in a transparent cylinder (diameter 20 cm and height 45 cm). The times of touching the wall by the forelimb of rats were recorded. The detailed operation was described previously [22].

#### The modified grip-traction test

Rats were hung to a horizontal rope (a 0.6-cm-diameter plastic tube placed horizontally 45 cm above the table) by its forepaws to evaluate the muscle strength [23]. Time of fall (maximum 60 s) was noted.

### Tissue preparation

Rats were anesthetized with ketamine and xylazine in 0.9% saline at 4, 7, and 28 days after MCAO and perfused with 0.9% saline, followed by fresh cold 4% PFA in 0.9% saline. Brain tissues were fixed in fresh 4% PFA 4 °C overnight and dehydrated gradient in 20% and 30% sucrose at 4 °C before sectioning on a Leica-1950 cryostat (Leica Instruments, Germany) at 10 μm and 30 μm. Sections were stored at − 20 °C for immunohistochemistry.

### Cresyl violet staining

Cerebral infarct volume was detected at 4 days, 7 days, and 28 days after MCAO [24]. Rats were anesthetized and perfused with physiological saline followed by 4% paraformaldehyde (PFA) at 4 days, 7 days, and 28 days after MCAO. The brains were removed carefully and fixed in PFA for 24 h, then dehydrated in 20% and 30% sucrose for 24 h respectively in 4 °C. The brain tissue was cut serially at 30 μm in coronal plan. The sections were stained by cresyl violet.

### Immunofluorescence

Brain sections were permeabilized by 0.3% Triton-100 in PBS, blocked by 10% goat serum in 90% PBS at room temperature for 1 h, and then incubated primary antibody at 4 °C overnight. The primary antibodies were as follows: mouse anti-CD200R (1:50, Bio-Rad), mouse anti-CD68 (1:300, Bio-Rad), and anti-Iba1 (1:300, Wako, Japan). After rinsed three times, sections were incubated with Alexa Flour 488 conjugated goat anti-rabbit IgG (1:500, Invitrogen) and Alexa Flour 633 conjugated goat anti-mouse IgG (1:1000, Invitrogen) at room temperature for 1 h. The fluorescent imaging was collected by an Olympus fluorescence microscope and processed by ImageJ software.

### Golgi-Cox staining

Animals were sacrificed and brain tissues were removed at 28 days after MCAO. Samples were immersed in Golgi-Cox solutions for 2 weeks and then dehydrated gradient in 20% and 30% sucrose at 4 °C. Tissues were cut into 100-μm sections by Leica-1950 cryostat (Leica Instruments, Germany) for following staining. Sections were immersed in 75% ammonia solution and 1% sodium thiosulfate. Images were collected on a microscope, and 10~20 neurons per sample were randomly observed for morphological analysis.

### BV2 cell culture and oxygen–glucose deprivation (OGD)

Cells from the immortalized mouse microglia cell line BV-2 were cultured in DMEM containing 10% fetal bovine serum (FBS) and maintained at 37 °C in 5% CO_2_ and 95% oxygen. BV2 were treated with IgG (15 μg/ml) or CD200Fc (15 μg/ml) 30 min before OGD. BV2 were subjected to OGD as previously reported [25, 26] with a slight modification. For OGD induction, BV2 culture medium was replaced with OGD buffer that contained 4.09 g NaCl, 186.5 mg KCl, 111 g CaCl_2_, 5 ml HEPES, and 1.125 g glycine in a total volume of 500 ml. Then, BV2 were kept in a humidified atmosphere containing 95% nitrogen and 5% CO_2_ for 6 h and reoxygenation for 16 h. For reoxygenation, OGD buffer was replaced with BV2 culture medium and cells were returned to normoxic conditions (95% air and 5% CO2). BV2 cultured under the normoxic condition served as the control.

To determine whether CD200Fc could inhibit JNK and P38 phosphorylation in BV2 cell, CD200Fc (15 μg/ml) or IgG (15 μg/ml) in the presence of anisomycin (1 nM) was added to the BV2 for 30 min, and BV2 cultured under the normoxic condition served as the control.

### Real-time quantitative RT-PCR (RT-qPCR)

At 4, 7, and 28 days after MCAO, brain samples were collected from ipsilateral sensorimotor cortex. Total RNA was isolated from frozen tissues using TRIzol Reagent (Vazyme, Nanjing, China) according to the manufacturer’s protocol. Equal amounts of total RNA were reverse transcribed under standard condition using HiScript 1st Strand cDNA Synthesis Kit (Vazyme, Nanjing, China). Quantitative PCR was performed on an ABI7000 real-time PCR system (Applied Biosystems, Inc., University Park, IL, USA) using SYBR Green Master Mix (Vazyme, Nanjing, China). The cycle time values were normalized to GAPDH of the same sample. Primer sequences are shown in Table 1.

**Table 1:** Primer sequences

Gene	Forward primer (5′–3′)	Reverse primer (5′–3′)
*Il-1β*	TCCAGGATGAGGACCCAAGC	TCGTCATCATCCCACGAGTCA
*Il-6*	CAGGAACGAAAGTCAACTCCA	ATCAGTCCCAAGAAGGCAACT
*Tnf-α*	TTCCCAAATGGGCTCCCTCT	GTGGGCTACGGGCTTGTCAC
*Il-4*	CAGGGTGCTTCGCAAATTTTAC	ACCGAGAACCCCAGACTTGTT
*Cd206*	GGTTCCGGTTTGTGGAGCAG	TCCGTTTGCATTGCCCAGTA
*Gapdh*	CAGCCTCGTCTCATAGACAAGATG	AAGGCAGCCCTGGTAACCA

### Western blot

At 4, 7, and 28 days after MCAO, brain samples were collected from the ipsilateral sensorimotor cortex. Brain tissues were homogenized with RIPA lysate (Beyotime, Nanjing, China) supplied with protease inhibitor cocktail (Roche, Indianapolis, IN, USA). An equal amount of protein was separated on SDS polyacrylamide gels, transferred to polyvinyl-difluoride membranes (Millipore, Billerica, MA, USA), and blocked with 2.5% (w/v) bovine serum albumin (BSA). The membrane was incubated with primary antibody (mouse anti-CD200R, 1:500, Bio-Rad, USA goat anti-CD200, 1:2000, RD, USA; rabbit anti-p-P65, 1:1000, CST, USA; rabbit anti-P65, 1:1000, CST, USA; rabbit anti-p-ERK, 1:1000, CST, USA; rabbit anti-ERK, 1:1000, CST, USA; rabbit anti-p-JNK, 1:1000, CST, USA; rabbit anti-JNK, 1:1000, CST, USA; rabbit anti-p-P38, 1:1000, CST, USA; rabbit anti-P38, 1:1000, CST, USA; mouse anti-actin, 1:1000, Santa Cruz Biotechnology Inc, USA) at 4 °C overnight. After being rinsed three times (7 min/time) with TBST, the membrane was incubated corresponding secondary antibody at room temperature for 1 h. After washed, the labeled proteins were detected using the Bio-Rad system (Bio-Rad, Germany).

### Statistical analysis

**Fig. 1:**
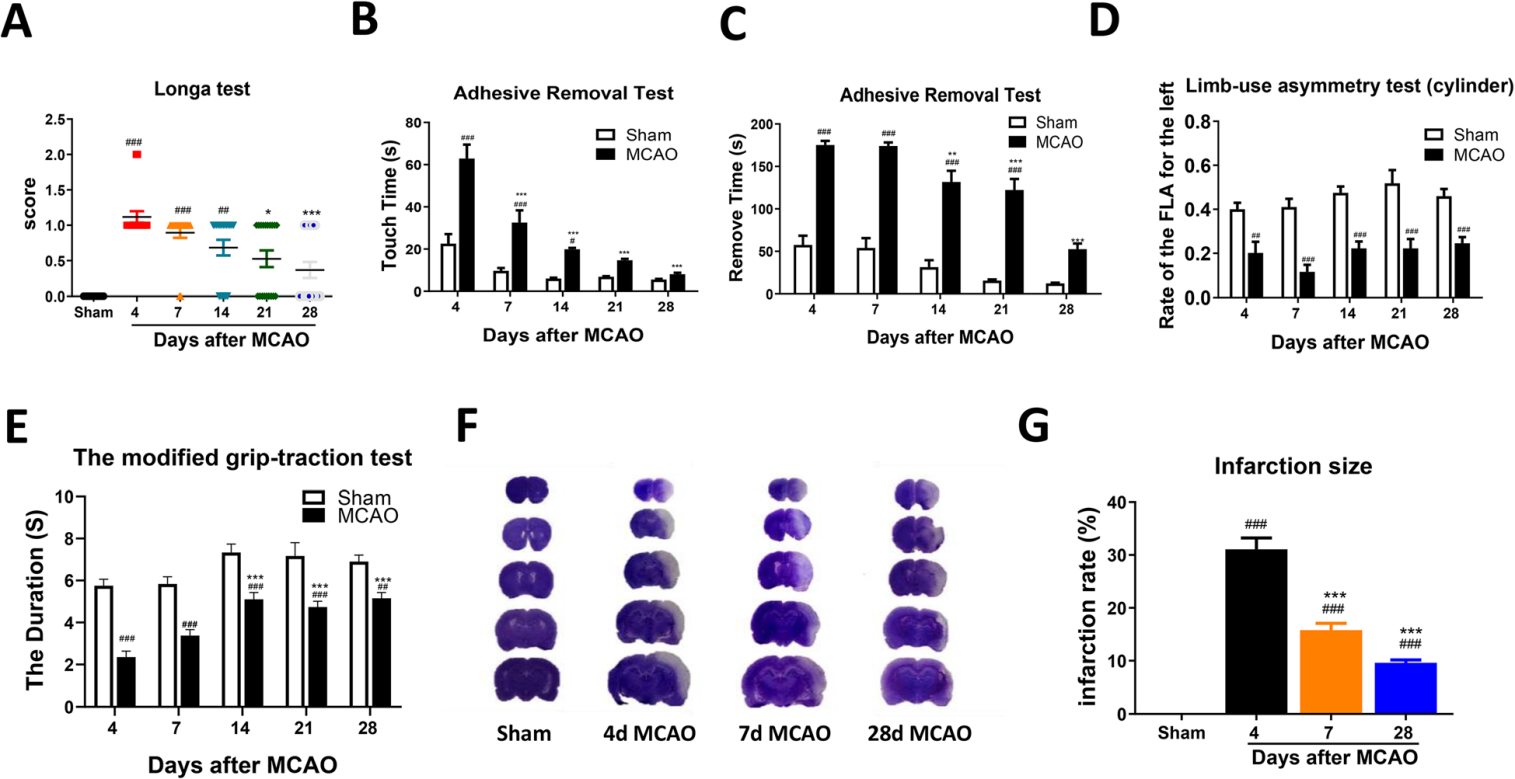
Change of sensorimotor functions and brain infarct volume after ischemic stroke in rats. Neurological functional change after MCAO in rats as shown in Longa test (**a**). Sensorimotor functional change after MCAO in rats as evaluated by adhesive removal test (**b, c**), Limb-use asymmetry test (cylinder) (**d**) and modified grip-traction test (**e**). Change of brain infarct volume after MCAO was assessed by cresyl violet staining (**f, g**). *n* = 8–15, results are expressed as means ± SEM. ^###^*P* < 0.001, ^##^*P* < 0.01, ^*#*^*P* < 0.05 versus the Sham group. ^***^P < 0.001, ^**^P < 0.01, ^*^P < 0.05 versus the 4 days MCAO group

The GraphPad Prism Software 6 (La Jolla, CA, USA) was used for statistical analysis, and the results were presented as a mean ± SEM. Among the frequencies, Longa test (Fig. 1a and Fig. 4a) was analyzed by non-parametric Mann-Whitney test. Adhesive removal test, limb-use asymmetry test (cylinder), and the modified grip-traction test (Fig. 1b–e and Fig. 4b–e) were performed by using two-way analysis of variance (ANOVA) with post hoc Student-Newman-Keuls test. For experiments with more than two groups and two factors of the parameters, results were compared using two-way analysis of variance (ANOVA) followed by post hoc Student-Newman-Keuls test. All tests were considered statistically significant at *P <* 0.05.

## Results

### Sensorimotor functional spontaneous recovery after stroke in rats

Sensorimotor functions were first assessed by performing a series of behavioral tests after MCAO surgery. Briefly, the Longa test for neurological disorders was performed (Fig. 1a), and sensorimotor dysfunction was identified using adhesive removal, limb-use asymmetry, and grip-traction tests (Fig. 1b–e). MCAO-induced neurological disorder observed at 4, 7, and 14 days improved gradually at 21 and 28 days compared with 4 days after MCAO (*P <* 0.05) (Fig. 1a). In the adhesive removal test, rats spent more time touching the spot at 4, 7, and 14 days after MCAO (time main effect, *F*_4, 110_ = 8.819, *P <* 0.0001) and spent more time removing the spot at 4, 7, 14, and 21 days after MCAO compared with the sham group (time main effect, *F*_4, 110_ = 4.222, *P =* 0.0032) (Fig. 1b, c). The time taken to touch or remove the spot decreased gradually as spontaneous recovery proceeded compared with 4 days (*t*-test, *P <* 0.05) (Fig. 1b, c). The injured forelimb mobility of MCAO rats was visibly reduced at each time point after MCAO, as determined by the limb-use asymmetry test (time main effect, *F*_4, 121_ = 0.6806, *P =* 0.6067) (Fig. 1d). The hanging time was significantly reduced at 4, 7, 14, 21, and 28 days in the MCAO group compared with the sham group, and partial recovery occurred at 14, 21, and 28 days after MCAO compared with 4 days (*t*-test, *P <* 0.05) (Fig. 1d).

When the size of the cerebral infarction was assessed by cresyl violet staining, the size decreased over time (*F*_3, 24_ = 101.0, *P <* 0.0001) (Fig. 1f, g). Taken together, these data suggest that neurological dysfunction occurred immediately after MCAO, followed by slow and limited spontaneous recovery.

### Expression of synapse-related proteins and synaptic loss after cerebral ischemia in rats

**Fig. 2:**
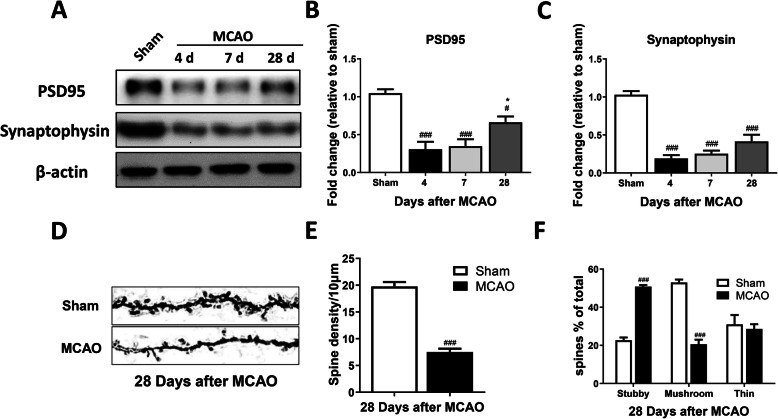
The expression of synapse-related proteins after ischemic stroke in rats. The protein levels of PSD95 and SYP were downregulated after MCAO in ipsilateral sensorimotor cortex (**a–c**). Dendritic spines density and morphology were detected by Golgi-Cox staining at 28 days after MCAO (**d–f**) (*n* = 4, scale bar: 5 μm). *n* = 3, results are expressed as means ± SEM. ^###^*P* < 0.001, ^##^*P* < 0.01, ^*#*^*P* < 0.05 versus the Sham group. ^***^P < 0.001, ^**^P < 0.01, ^*^P < 0.05 versus the 4 days MCAO group

Synaptic plasticity plays an important role in functional recovery after stroke [27, 28], and synapse-related proteins and dendritic spine density are important indices of synaptic plasticity. Thus, we measured the protein levels of postsynaptic density protein 95 (PSD-95) and synaptophysin (SYP) in the ipsilateral sensorimotor cortex. The protein levels of PSD95 (*F*_3, 8_ = 17.51, *P =* 0.0007) and SYP (*F*_3, 8_ = 43.22, *P <* 0.0001) were decreased significantly at 4, 7, and 28 days after MCAO compared with the sham group (Fig. 2a–c). However, compared with the 4-day MCAO group, the expression of PSD95 was increased markedly at 28 days after MCAO (*t*-test, *P <* 0.05) (Fig. 2a, b). When dendritic spine density and morphology in the ipsilateral sensorimotor cortex were measured, dendritic spine density was reduced dramatically (*t*-test, *P <* 0.0001), the percent stubby spine was increased significantly, and the percent mushroom spine was decreased significantly at 28 days after MCAO (group main effect, *F*_2, 12_ = 66.62, *P <* 0.0001) (Fig. 2d–f). These data suggest the presence of markedly decreased synaptic function after stroke and slow recovery following the initial event.

### The expression of CD200 and CD200R after cerebral ischemia in rats

**Fig. 3:**
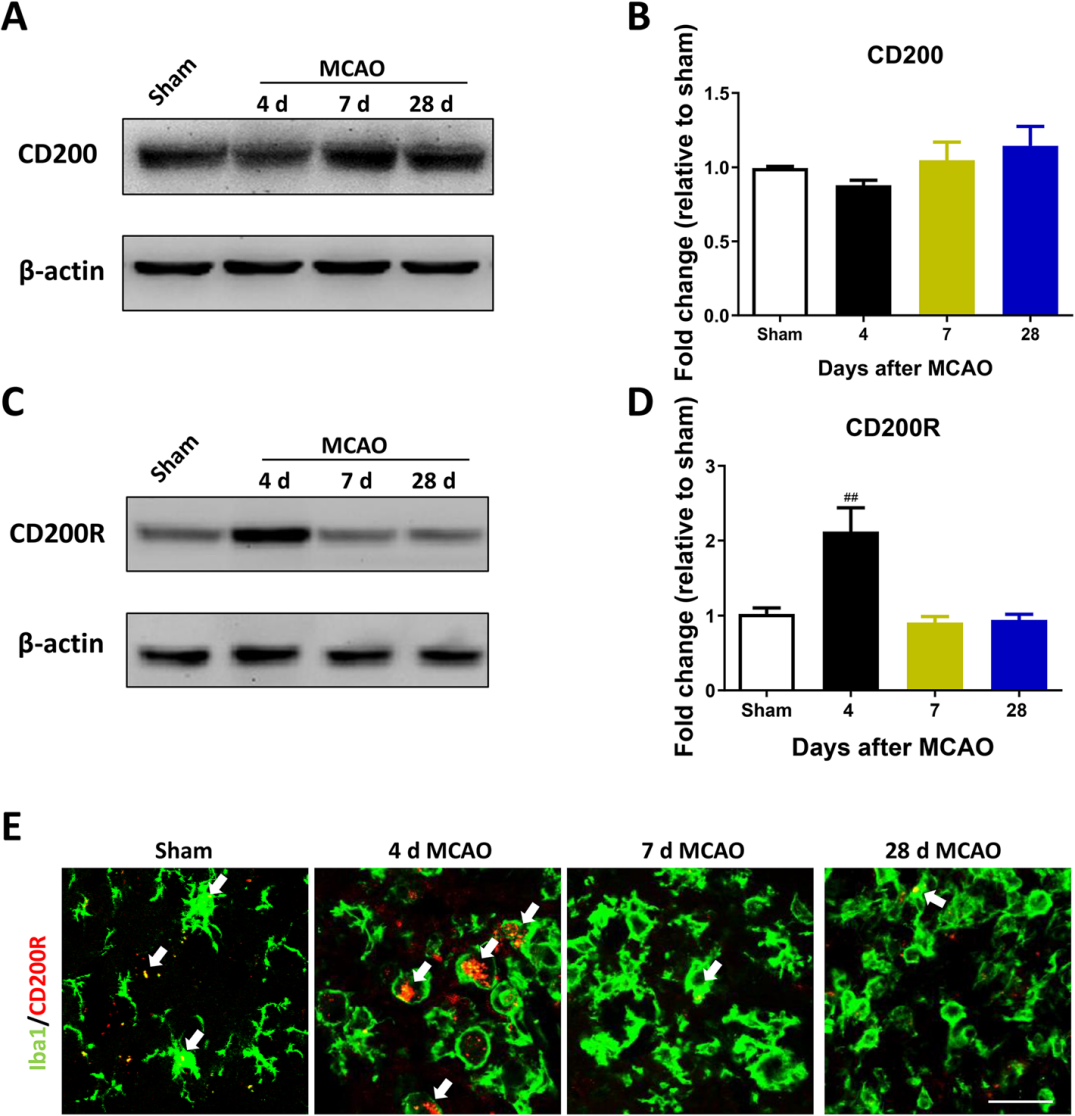
CD200R was transiently upregulated after ischemic stroke in rats. Representative western blots of CD200, CD200R, and their corresponding normalized control β-actin in ipsilateral sensorimotor cortex at 4 days, 7 days, and 28 days after MCAO (**a**, **c**). The levels of CD200R was upregulated at 4 days after MCAO, but MCAO did not influence the expression of CD200 at each time point (**b, d**). CD200R (red) staining showed protein expression at 4 days and colocalized with Iba1^+^(green) cells (Iba1: a microglia marker) after MCAO in ipsilateral sensorimotor cortex (**e**) (*n* = 3, Scale bar: 20 μm). Results are expressed as means ± SEM. ^###^*P* < 0.001, ^##^*P* < 0.01, ^*#*^*P* < 0.05 versus the Sham group

Based on the important role of the CD200/CD200R signaling pathway on regulating synaptic plasticity in many pathological conditions [16, 29, 30], we investigated the expression of CD200 and CD200R in the ipsilateral sensorimotor cortex. There were no obvious differences in the expression of CD200 between the sham and MCAO groups (group main effect, *F*_3, 8_ = 1.468, *P =* 0.2947) (Fig. 3a, b). However, the expression of CD200R was increased markedly at 4 days after MCAO compared with the sham group (group main effect, *F*_3, 8_ = 11.36, *P =* 0.0030) (Fig. 3)c, d. Immunofluorescent staining showed that CD200R was expressed on Iba1-positive cells (Fig. 3e). These results suggest that the CD200/CD200R signaling pathway may be involved in spontaneous functional recovery after stroke onset in rats.

### Functional outcomes after CD200/CD200R signaling pathway modification post-cerebral ischemia in rats

**Fig. 4:**
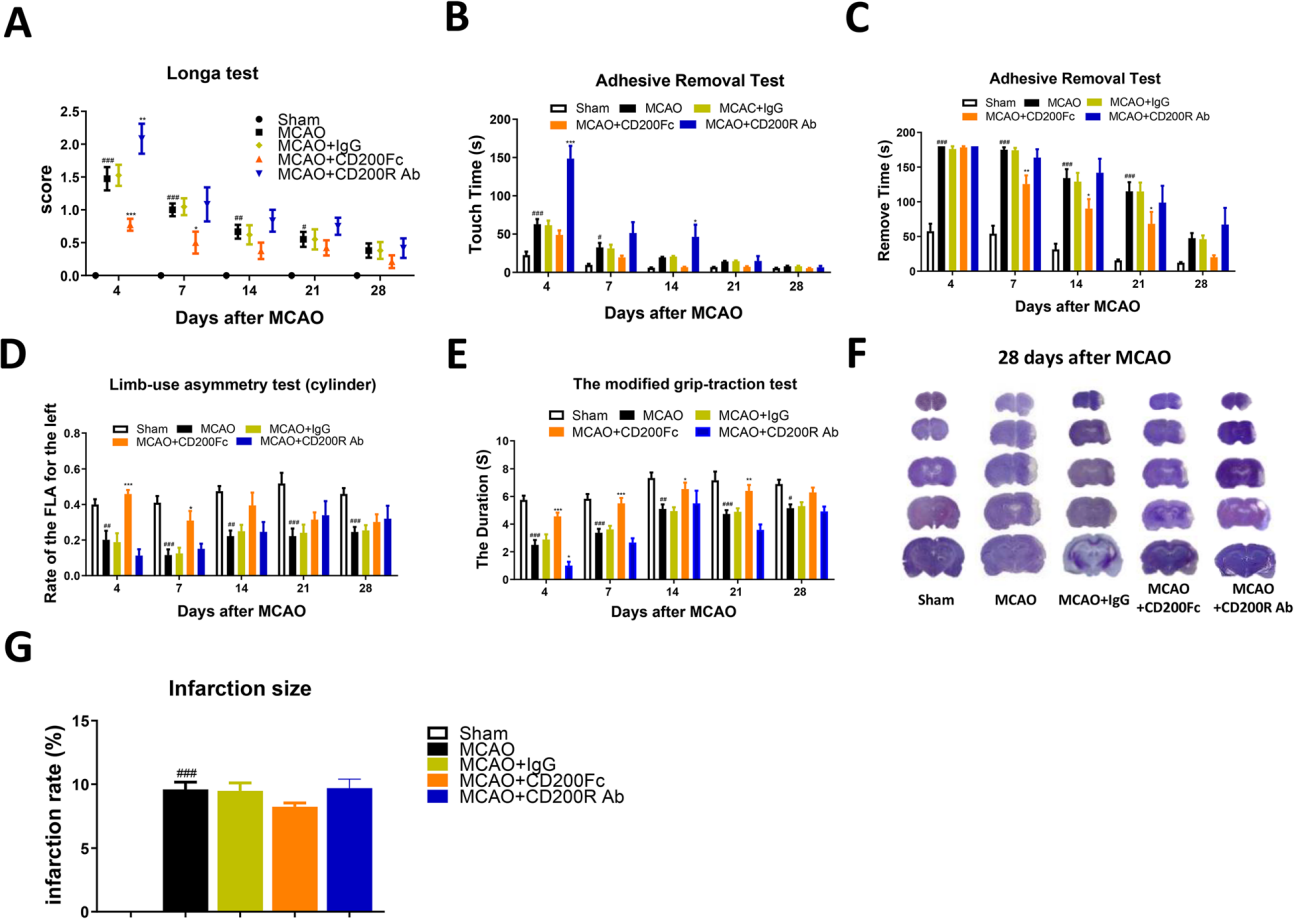
CD200/CD200R pathway contributed to functional recovery after ischemic stroke in rats. Sensorimotor functions were detected by Longa test (**a**), adhesive removal test (**b**, **c**), limb-use asymmetry test (cylinder) (**d**), and modified grip-traction test (**e**) after CD200Fc or CD200R Ab i.c.v injection, *n* = 8–15. The sizes of cerebral infarction were detected by cresyl violet staining (**f**, **g**), *n* = 7. Results are expressed as means ± SEM. ^###^*P* < 0.001, ^##^*P* < 0.01, ^*#*^*P* < 0.05 versus the Sham group. ^***^P < 0.001, ^**^P < 0.01, ^*^P < 0.05 versus the MCAO+IgG group

To determine whether modulating CD200/CD200R signaling affects spontaneous functional recovery, we performed a battery of behavioral tests to evaluate sensorimotor function after intracerebroventricular (i.c.v.) injection with CD200 fusion protein (CD200Fc) or CD200R blocking antibody (CD200R Ab) post-stroke. First, CD200Fc significantly improved neurological recovery in Longa tests at 4 and 7 days after MCAO, whereas neurological recovery was restrained at 4 days after CD200R Ab injection compared with the MCAO+IgG group (*P <* 0.05) (Fig. 4a). Second, sensorimotor functions were assessed using adhesive removal, limb-use asymmetry, and modified grip-traction tests. Rats spent more time touching the spot at 4 and 14 days in the MCAO+CD200R Ab group (group main effect, *F*_4, 312_ = 37.42, *P <* 0.0001) (Fig. 4b), whereas it took less time to remove the spot at 7, 14, and 21 days in the MCAO+CD200Fc group compared with the MCAO+IgG group (group main effect, *F*_4, 312_ = 44.91, *P <* 0.0001) (Fig. 4c). CD200Fc increased the frequency of injured forelimb use at 4 and 7 days after MCAO as determined by the limb-use asymmetry test (group main effect, *F*_4, 292_ = 29.75, *P <* 0.0001) (Fig. 4d). In the modified grip-traction test, the time spent hanging onto the rope was increased markedly at 4, 7, 14, and 21 days after MCAO in the MCAO+CD200Fc group compared with the MCAO+IgG group. In contrast, CD200R Ab administration decreased the time spent hanging onto the rope at 4 days after MCAO (group main effect, *F*_4, 364_ = 47.51, *P <* 0.0001) (Fig. 4e). We also detected the effect of CD200/CD200R signaling on the size of cerebral infarction. The result shown that CD200/CD200R signaling pathway modification did not influence the size of cerebral infarction at 28 days after MCAO (group main effect, *F*_4, 30_ = 66.06, *P <* 0.0001) (Fig. 4f, g). These data suggest that activation of the CD200/CD200R signaling pathway promoted spontaneous functional recovery after stroke in rats.

### Synaptic plasticity changes in cerebral ischemia rats after CD200/CD200R signaling pathway alteration

**Fig. 5:**
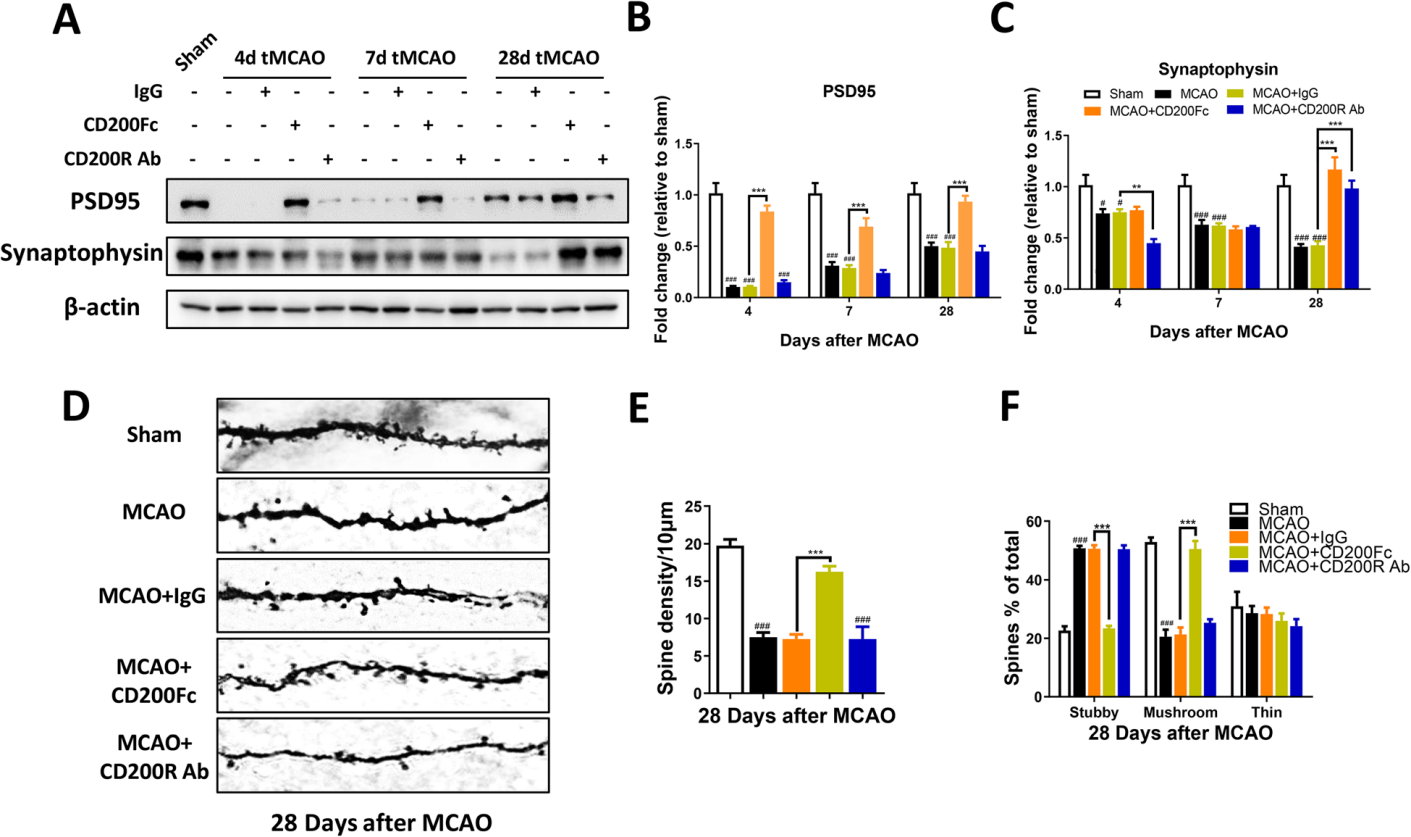
Activating CD200/CD200R pathway upregulated synapse-related proteins expression after ischemic stroke in rats. Representative western blots of PSD-95 and synaptophysin (SYP) and their corresponding normalized control β-actin ipsilateral sensorimotor cortex at 4 days, 7 days, and 28 days after MCAO (**a**). CD200Fc increased the protein levels of PSD-95 at 4, 7, and 28 days and the protein levels of SYP at 28 days after MCAO in ipsilateral sensorimotor cortex (**b**, **c**) (*n* = 3). CD200R Ab reduced the protein levels of SYP at 4 days after MCAO in ipsilateral sensorimotor cortex (**c**) (*n* = 3). Dendritic spines’ density and morphology were detected by Golgi-Cox staining at 28 days after MCAO (**d–f**) (*n* = 4, scale bar: 5 μm). Results are expressed as means ± SEM. ^#^*P* < 0.05, ^##^*P* < 0.01, ^###^*P* < 0.001 versus the Sham group. ^**^P < 0.05, ^**^P < 0.01, ^***^P < 0.001 versus the MCAO+IgG group

Neuronal connectivity and synaptic plasticity are essential to achieve complex tasks such as sensorimotor behavior. Thus, we sought to determine whether the CD200/CD200R signaling pathway participates in regulating synaptic plasticity after MCAO. Data from Western blotting demonstrated that the protein level of PSD-95 was increased significantly at 4, 7, and 28 days (group main effect, *F*_4, 30_ = 101.1, *P <* 0.0001), and SYP levels were increased at 28 days (group main effect, *F*_4, 30_ = 23.64, *P <* 0.0001) after CD200Fc i.c.v injection post-stroke (Fig. 5a–c). In contrast, the expression of SYP was reduced markedly at 4 days after CD200R Ab injection post-stroke (group main effect, *F*_4, 30_ = 23.64, *P <* 0.0001) (Fig. 5a, c). Moreover, the stroke-induced reduction in spine density was rescued (group main effect, *F*_4, 15_ = 36.91, *P <* 0.0001), and spine shape was altered from stubby to mushroom by CD200Fc application at 28 days after MCAO (group main effect, *F*_4, 30_ = 0.5636, *P =* 0.6909) (Fig. 5d–f). These results suggest that CD200Fc may enhance synaptic plasticity, thereby promoting sensorimotor functional recovery.

### Microglial response and inflammatory factor release after CD200/CD200R signaling pathway modification in cerebral ischemia rats

**Fig. 6:**
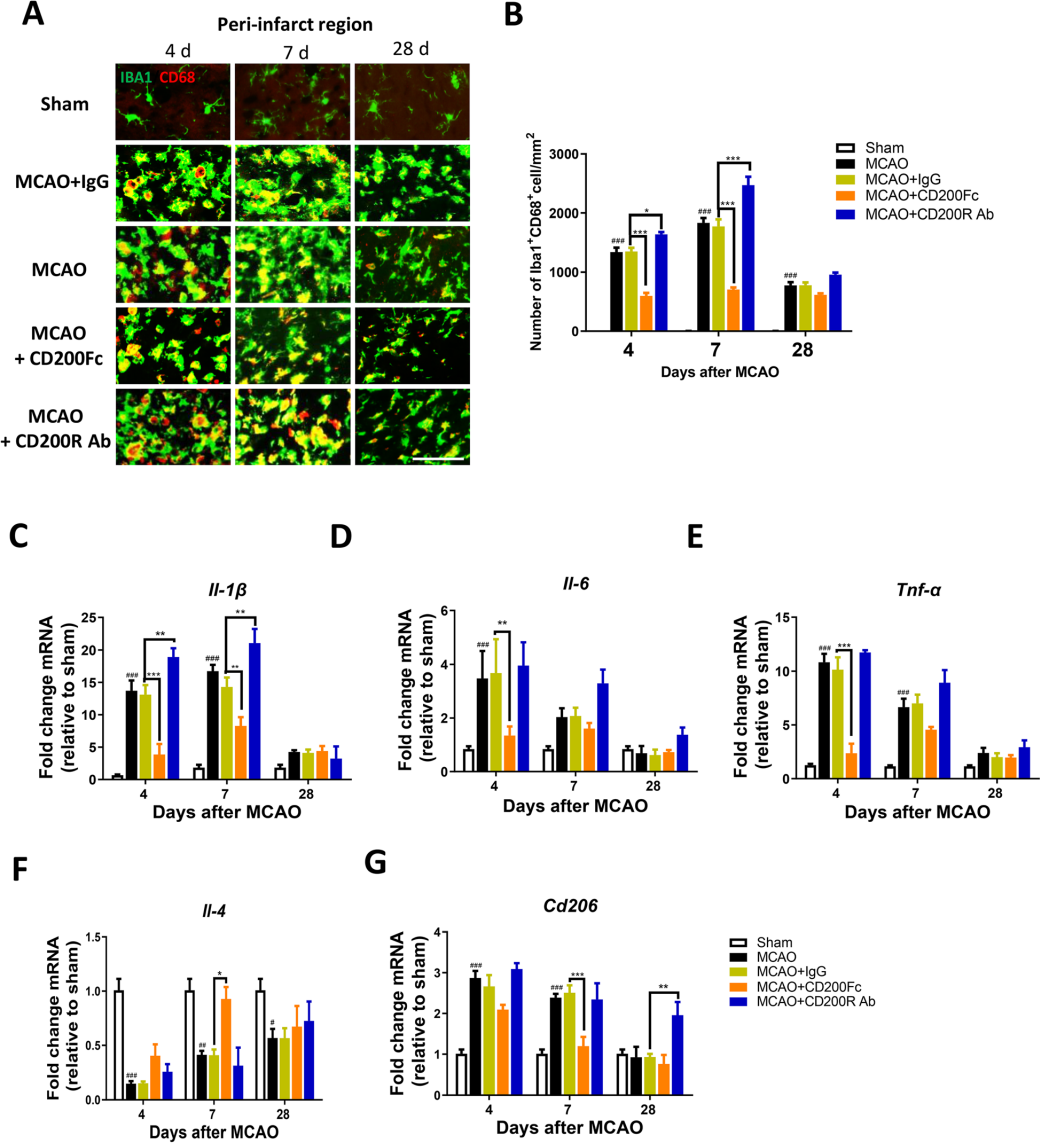
Microglia activation and inflammatory release were inhibited via activating the CD200/CD200R pathway after ischemic stroke in rats. Representative sections of brain in different groups were immunostained with iba-1 (green) and CD68 (red, marker of microglia activation) in ipsilateral sensorimotor cortex after MCAO (**a**) (*n* = 3, scale bar: 50 μm), CD200Fc decreased the number of iba-1^+^/CD68^+^ cells at 4 and 7 days compared with MCAO+IgG group, whereas CD200R Ab reversed the effect. The effect of CD200/CD200R signaling pathway on the mRNA levels of *Il-1β*, *Il-6*, *Tnf-α*, *Il-4*, and *Cd206* in ipsilateral sensorimotor cortex (**c–g**), (*n* = 4–5). Results are expressed as means ± SEM. ^#^*P* < 0.05, ^##^*P* < 0.01, ^###^*P* < 0.001 versus the Sham group. ^**^P < 0.05, ^**^P < 0.01,^***^P < 0.001 versus the MCAO+IgG group

Synaptic plasticity is impaired by exaggerated inflammatory responses and microglia activation during central nervous system (CNS) injury and neurodegenerative diseases [11, 31], and the CD200/CD200R signaling pathway plays an important role in regulating microglia activation and inflammatory factor release [32–34]. To investigate whether the CD200/CD200R signaling pathway is involved in regulating microglia activation and inflammatory factor release after stroke in rats, we assessed microglia activation using immunofluorescence and inflammatory factor (such as *Il-1β*, *Il-6*, *Tnf-α*, *Il-4*, and *Cd206*) release. The number of Iba1^+^/CD68^+^ cells in the ipsilateral sensorimotor cortex was decreased markedly at 4 and 7 days after MCAO in the MCAO+CD200Fc group compared with the MCAO+IgG group (group main effect, *F*_4, 30_ = 313.5, *P <* 0.0001) (Fig. 6a, b). In contrast, CD200R Ab significantly increased the number of Iba1+/CD68+ cells at 4 and 7 days after MCAO (group main effect, *F*_4, 30_ = 313.5, *P <* 0.0001) (Fig. 6a, b). The mRNA levels of *Il-1β*, *Il-6*, *Tnf-α*, and *Cd206* were downregulated, whereas *Il-4* was upregulated, following CD200Fc application after MCAO (*Il-1β* at 4 and 7 days after MCAO; *Il-6* and *Tnf-α* at 4 days after MCAO; *Cd206* at 7 days after MCAO; *Il-4* at 7 days after MCAO) (*P <* 0.05) (Fig. 6c–g). In contrast, CD200R Ab increased the mRNA levels of *Il-1β* at 4 and 7 days after MCAO and of *Cd206* at 28 days (*P <* 0.05) (Fig. 6a, g). These observations suggest that activation of the CD200/CD200R signaling pathway could ameliorate microglia activation and inflammatory factor release after stroke in rats.

### The effect of CD200/CD200R signaling pathway modification on NF-*κ*B and p-MAPK expression after cerebral ischemia in rats

**Fig. 7:**
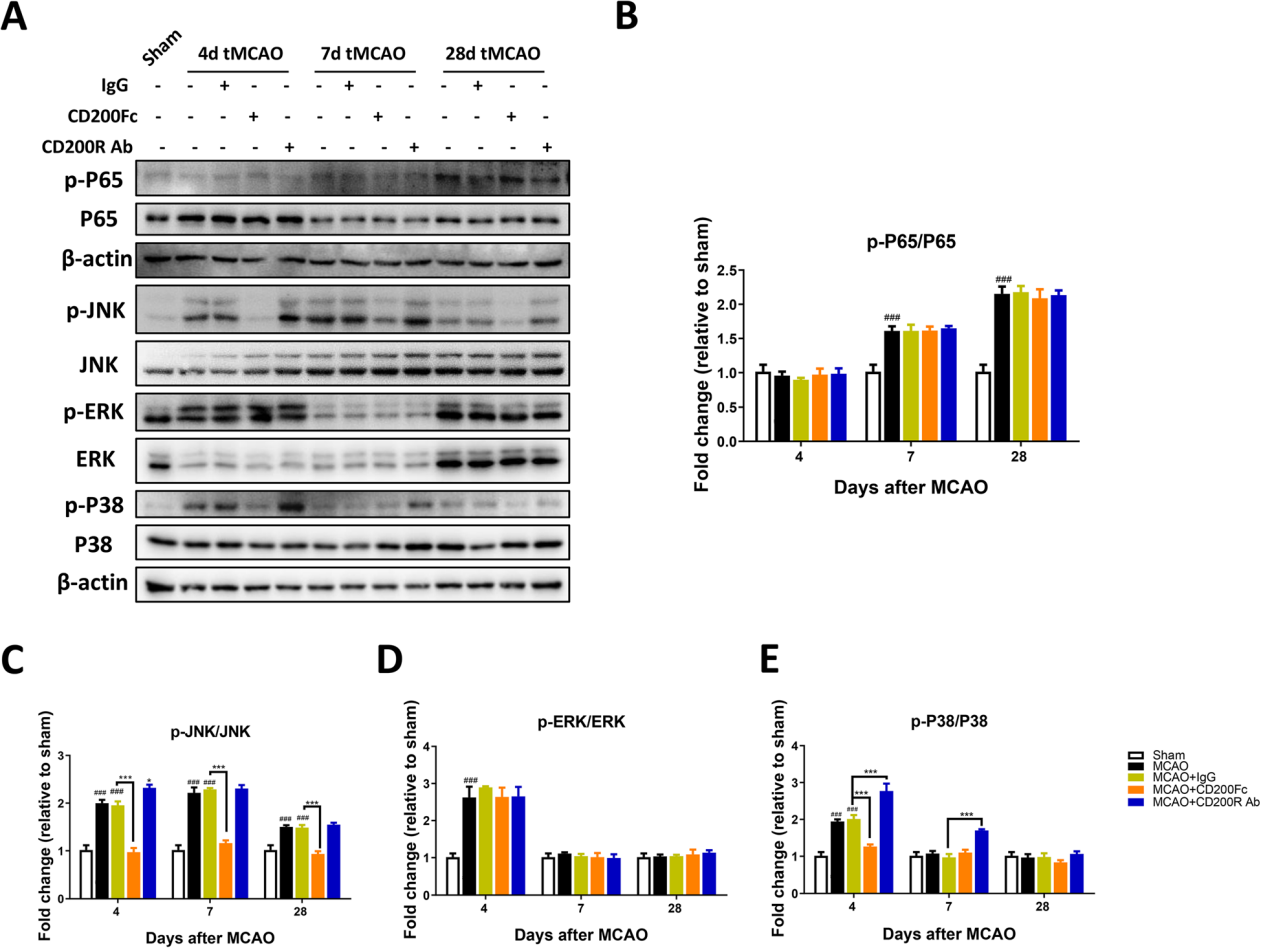
The effect of CD200/CD200R pathway on NF-*κ*B and MAPK-related proteins expression after ischemic stroke in rats. CD200/CD200R signaling pathway unaffected the phosphorylation of NF-*κ*B (**a, b**) and ERK (**a**, **d**). CD200Fc inhibited phosphorylation of JNK (**a**, **c**) and P38 (**a**, **e**), *n* = 3. Results are expressed as means ± SEM. ^#^*P* < 0.05, ^##^*P* < 0.01, ^###^*P* < 0.001 versus the Sham group. ^**^P < 0.05, ^**^P < 0.01, ^***^P < 0.001 versus the MCAO+IgG group

Previous studies showed that activating the CD200/CD200R signaling pathway limited microglia activation and inflammatory responses by inhibiting the NF-*κ*B and mitogen-activated protein kinase (MAPK) pathways [35, 36]. To further investigate the mechanism through which CD200/CD200R signaling regulates synaptic plasticity after stroke, we assessed the expression of p-p65, phospho-c-Jun N-terminal kinase (p-JNK), phospho-extracellular signal-regulated kinase-1/2 (p-ERK1/2), and phospho-protein 38 (p-p38) MAPKs in the ipsilateral sensorimotor cortex. P65 phosphorylation was observed in the MCAO group at 7 and 28 days compared with the sham group (group main effect, *F*_4, 30_ = 26.20, *P <* 0.0001) (Fig. 7a, b), but the CD200/CD200R signaling pathway did not influence p65 phosphorylation at each time point after MCAO. The protein levels of p-JNK, p-ERK, and p-p38 were increased after MCAO compared with the sham group (p-JNK at 4, 7, and 28 days after MCAO, group main effect, *F*_4, 30_ = 141.5, *P <* 0.0001; p-ERK at 4 days after MCAO, group main effect, *F*_4, 30_ = 10.64, *P <* 0.0001; p-p38 at 4 and 7 days after MCAO, group main effect, *F*_4, 30_ = 36.43, *P <* 0.0001) (Fig. 7a, c–e). The protein levels of p-JNK and p-p38 were decreased significantly by CD200Fc (p-JNK at 4, 7, and 28 days after MCAO; p-p38 at 4 days after MCAO) (Fig. 7a, c, e). CD200R Ab increased the expression of p-JNK at 4 days and p-p38 at 4 and 7 days after MCAO compared with the MCAO+IgG group (Fig. 7a, c, e). This suggests that activating the CD200/CD200R signaling pathway downregulates p-MAPK expression after stroke in rats.

### CD200Fc inhibited JNK and P38 phosphorylation after OGD in BV2 microglia cell line

**Fig. 8:**
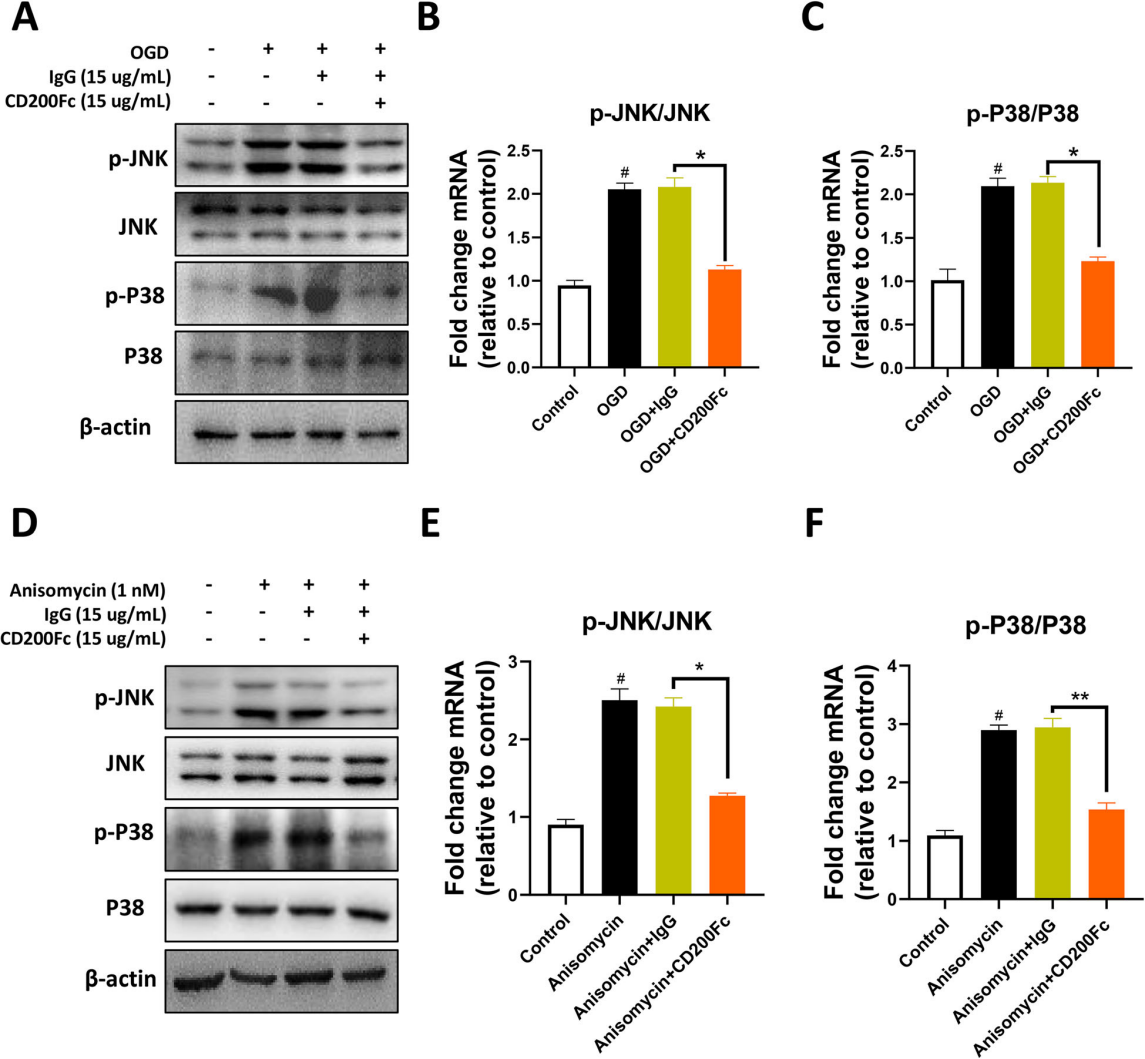
JNK and P38 phosphorylation were restrained by CD200Fc after OGD in BV2 cells. CD200Fc inhibited phosphorylation of JNK **(a**, **b)** and P38 **(a**, **c**). Results are expressed as means ± SEM. ^#^*P* < 0.05, ^##^*P* < 0.01, ^###^*P* < 0.001 versus the control group. ^**^P < 0.05, ^**^P < 0.01, ^***^P < 0.001 versus the OGD+IgG group. CD200Fc reversed the phosphorylation of JNK (**d**, **e**) and P38 which induced by anisomycin (an agonist of JNK and P38) (**d**, **f**), *n* = 3. Results are expressed as means ± SEM. ^#^*P* < 0.05, ^##^*P* < 0.01, ^###^*P* < 0.001 versus the control group. ^**^P < 0.05, ^**^P < 0.01, ^***^P < 0.001 versus the anisomycin+IgG group.

To test whether CD200Fc inhibited microglial activation is mediated via MAPKs especially JNK and p38, we used BV2 cells to test the effect of CD200Fc on JNK and P38 phosphorylation after OGD. As shown in Fig. 8a–c, JNK and P38 phosphorylation induced by OGD were reversed by CD200Fc (p-JNK, *F*_2, 6_ = 0.009141, *P* = 0.9909; p-P38, *F*_2, 6_ = 1.289, *P* = 0.3423). Next, we further determined the effect of CD200Fc on JNK and P38 phosphorylation in microglia via anisomycin (agonists of these JNK and P38), and the results showed that CD200Fc significantly decreased the protein level of p-JNK and p-p38 induced by anisomycin (p-JNK, *F*_2, 6_ = 3.756, *P* = 0.0876; p-P38, *F*_2, 6_ = 5.044, *P* = 0.0519). These data indicated that CD200Fc could inhibit JNK and p38 phosphorylation in microglia.

## Discussion

Recent studies indicated that the CD200/CD200R signaling pathway regulates the microglia activation-induced inflammatory response and synaptic plasticity in vitro and in vivo [32, 35, 37], but few studies have been performed in stroke. Here, we investigated CD200/CD200R as a potential target for promoting spontaneous recovery. The collected data support the viewpoint that sensorimotor deficiency was partially recovered by promoting synaptic plasticity. Activation of the CD200/CD200R signaling pathway further promoted functional recovery after stroke.

Ischemic stroke impairs the integrity of neural networks and intercellular signal transmission and causes sensorimotor deficits [6]. Surviving neural networks rewire and form new structural and functional circuits during spontaneous functional recovery [5]. PSD95 and SYP are crucial indicators of synaptic plasticity, which is involved in maturation of excitatory synapses and stabilization of synaptic contacts [38, 39]. Dendritic spine density is an important index of structural and functional plasticity, which is associated with rewiring of neuronal circuits; enhanced plasticity can contribute to recovery of cortical function [5, 40]. In the present study, the expression of PSD95 and dendritic spine density were increased significantly during spontaneous functional recovery, suggesting that neural networks were partially rewired after stroke.

The CD200/CD200R signaling pathway regulates synaptic plasticity, and dysfunction in this pathway contributes to synaptic deficits in aging and AD [18, 29]. In the current study, consistent with a previous report [41], CD200R expression was transiently increased in microglia after stroke. Activating the CD200/CD200R signaling pathway with CD200Fc remarkably increased PSD95 expression and dendritic spine density. In addition, the sensorimotor functional dysfunction induced by stroke was ameliorated by CD200Fc application post-stroke. These observations showed that activation of the CD200/CD200R signaling pathway improved recovery of sensorimotor function by facilitating the formation of new neural networks and protecting synaptic structures from ischemia-induced damage.

Inflammation and pro-inflammatory factors are secondary injury mediators following cerebral ischemia [42], and they exacerbate loss of dendritic spines [11]. Suppressing microglia activation (microgliosis) rescues local inflammatory over-release and improves outcomes after stroke [43, 44]. The CD200/CD200R signaling pathway helps modulate microglia activation and inflammatory factors in many injury models [33, 41]. In this study, regulating CD200/CD200R signaling using CD200Fc significantly inhibited microglial over-activation and profoundly influenced the microenvironment, including decreasing the mRNA levels of proinflammatory (*Il-1β*, *Il-6*, and *Tnf-α*) factors and increasing the mRNA levels of anti-inflammatory (*Il-4*) regulators in the ipsilateral cortex. Similar effects were also observed after CD200Fc application in LPS-stimulated microglia cells, during aging, and in Parkinson’s disease [18, 45]. These data suggest that CD200/CD200R signaling might help inhibit synapse loss after stroke by regulating the activation of microglia and modulating the balance between pro- and anti-inflammatory factors.

NF-*κ*B is an important transcription factor that promotes microglia activation and inflammatory factor release [46, 47]. Previous studies showed that CD200Fc could inhibit microglia activation and the inflammatory response by suppressing the NF-*κ*B pathway in vitro [48]. However, the current results showed that CD200/CD200R signaling had no effect on the NF-*κ*B pathway after stroke. CD200R is only expressed in microglia in the CNS, whereas inflammation is a complex response involving many kinds of cells. Activating CD200/CD200R signaling may be insufficient to contain the NF-κB pathway after stroke in vivo. Recent studies reported that the CD200/CD200R pathway modulated the inflammatory response by controlling MAPK activation in vitro and in vivo [49–51]. The current results showed that CD200Fc inhibited MAPK activation by suppressing the phosphorylation of p-JNK and p-p38 after stroke. This suggests that the effect of CD200/CD200R signaling on suppressing the inflammatory response may be exerted by inhibiting MAPK activation.

One limitation of the present study is that CD200R not only expressed in microglia, but also expressed in peripheral myeloid cells [52] and neuron in human according to the Ben Barres database. However, the infiltration of peripheral myeloid cells plays an important role in inflammatory response after stroke; CD200Fc or CD200R Ab may play a role in these cells. In this study, we did not distinguish between microglia and infiltrating myeloid cells, and CD200R deficient on microglia transgenic mice would be helpful to explore the role of CD200/CD200R signaling on microglia after stroke in the future studies.

## Conclusion

**Fig. 9:**
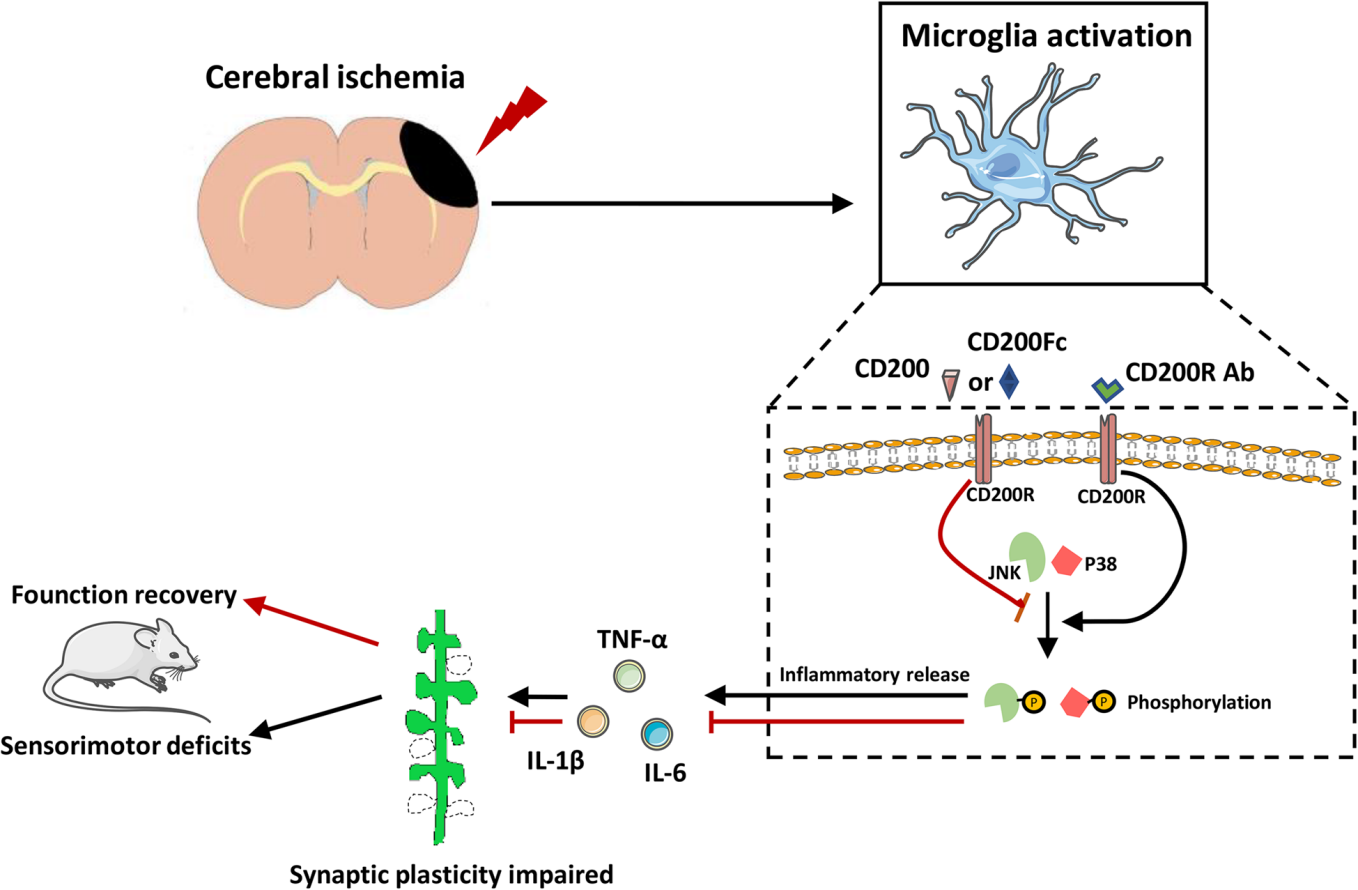
Schematic drawing of the effect of CD200/CD200R pathway promoting spontaneous functional recovery via inhibiting inflammatory response and enhancing synaptic plasticity after ischemic stroke

The CD200/CD200R signaling pathway participates in spontaneous functional recovery after stroke. Activation of CD200/CD200R signaling attenuated brain injury and facilitated the recovery of sensorimotor function. These effects were exerted by increasing synaptic plasticity via inhibiting microglia activation and improving the inflammatory microenvironment (Fig. 9). Therefore, the CD200/CD200R signaling pathway could be a potential therapeutic target for functional recovery after stroke.

## Data Availability

The manuscript included all relevant data.

## Abbreviations

MCAO: Transient middle cerebral artery occlusion
CD200R: CD200 receptor
CD200R1: CD200 receptor1
CD200Fc: CD200 fusion protein
CD200R Ab: CD200R blocking antibody
*Il-1β*: Interleukin 1 beta
*Tnf-α*: Tumor necrosis factor alpha
*Il-6*: Interleukin 6
*Il-4*: Interleukin 4
*Cd206*: Cluster of differentiation 206
LTP: Long-term potentiation
CBF: Cerebral blood flow
PSD-95: Postsynaptic density protein 95
SYP: Synaptophysin
CNS: Central nervous system
Iba1: Ionized calcium binding adapter molecule 1
NF-κB: Nuclear factor kappa-B
MAPK: Mitogen-activated protein kinase
p-JNK: Phospho-c-Jun N-terminal kinase
p-ERK1/2: Phospho-extracellular signal-regulated kinase-1/2
OGD: Oxygen–glucose deprivation
